# Enhanced specific immune responses by CpG DNA in mice immunized with recombinant hepatitis B surface antigen and HB vaccine

**DOI:** 10.1186/1743-422X-8-78

**Published:** 2011-02-23

**Authors:** Xiancheng Zhang, Peng He, Zhongyu Hu, Xingtai Wang, Zhenglun Liang

**Affiliations:** 1 The Second Division of Viral Vaccines; National Institute for the Control of Pharmaceutical and Biological Products; Beijing, PR China; 2 William A. Hinton State Laboratory Institute; Massachusetts Department of Public Health; Boston, the USA

## Abstract

**Background:**

Hepatitis B vaccine adjuvant, alum, is generally used for vaccination although it does not stimulate Th1 immunity and 10% of the population has low or no antibody response. Efforts have been continued to find more efficient vaccine adjuvants for better antibody response as well as stimulation of Th1 immunity.

**Methods:**

CpG DNA was used as an adjuvant for recombinant HBsAg to immunize 6- to 8-week-old female BALB/c mice with or without alum for different dosages. The production of HBsAb, CD80 and CD86 from dendritic cells, and cytokines IL-10, IL12, etc., were analyzed and compared for the performance of immunization.

**Results:**

5-20 μg CpG DNA had the best co-stimulation effect of HBsAb serum conversion for mice vaccinated with recombinant expressed HBsAg. The mice vaccinated with recombinant 20 μg CpG DNA and regular vaccine (containing alum adjuvant) had the highest concentration of antibody production. IL-12b, IL-12a and IL10 mRNA reached to the peak level between 3 and 6 hours after the CpG DNA induction in splenocytes. The expression levels of CD80 and CD86 leucocyte surface molecules were increased with 20 μg CpG DNA alone or with 20 μg CpG DNA and 4 μg HBsAg.

**Conclusions:**

Our results confirmed the adjuvant effect of CpG DNA for HBsAg in the mouse model. The increase of IL10 and IL12 production suggested the involvement of Th1 cell activation. The activation of CD80 and CD86 molecules by CpG-ODN might be part of the mechanism of T/B cells coordination and the enhancement of recombinant HBsAg induced immune response.

## Background

Hepatitis B (HB) is the disease caused by hepatitis B virus (HBV) infection and is the most commonly seen liver disease worldwide [1]. The virus can be transmitted parenterally, perinatally and sexually [2,3]. The viral infection has a worldwide distribution. The World Health Organization estimates that more than 2 billion people have been infected with HBV worldwide, 360 million of which are chronically infected and at risk of liver cirrhosis, liver cancer or other serious illness, or death. HBV is estimated to be responsible for 500, 000 to 700,000 deaths annually [4]. A survey study conducted by China CDC showed 7.2% weighted surface antigen (HBsAg) prevalence by ELISA among population aged 1~59 years old in 2006 [5].

Despite the progress in prophylaxis, diagnosis and treatment, vaccination is still the most cost effective way of fighting against the virus. The currently used recombinant HBsAg has to be combined with adjuvant, usually alum, due to the weak immunity production of the antigen alone. Alum has been used as vaccine adjuvant for more than 70 years, although the molecular mechanism of action and the target cells of alum are still unknown. It has been assumed that adsorption to alum increases antigen availability at the injection site, allowing an efficient uptake by antigen-presenting cells (APCs) [6]. The increased antigen uptake by dendritic cells (DCs) observed in vitro also supported the antigen delivery function [7]. The intraperitoneal (i.p.) injection of alum could induce the recruitment of monocytes, which may uptake the vaccine antigen [8]. Alum is generally recognized as a stimulator of Th2 immunity. However, it does not stimulate Th1 immunity and 10% of the population has low response or no antibody response [9].

CpG DNA can induce proliferation of almost all B cells and trigger polyclonal immunoglobulin (Ig) secretion, which is T cell independent and antigen nonspecific [10]. A strong synergetic response was observed when the CpG DNA is used together with alum [11]. We show the dose related enhancement effect of CpG DNA in increasing the production of HBsAb in mouse compared with the recombinant antigen of HBsAg alone or alum adjuvant HBV vaccine. The synergetic response was also observed in the expression levels of cluster of differentiation 80 (CD80) and cluster of differentiation 86 (CD86). The dynamics of a group of cytokines was analyzed and compared for different experiment groups.

## Methods

### Oligodeoxynucleotides

ODN (BW006) was provided by Yunnan Wosen Biotechnology Company. BW006 was synthesized with the sequence of 5'-tcgacgttcgtcgttcgtcgttc-3' and followed by sulphurization. Lipopolysaccharide (**LPS**) level in BW6 was less than 0.5 ng/mg by Limulus assay.

### Recombinant HBsAg

The awd2 subtype HBsAg was expressed in Hansenular polymorpha yeast and produced by Yunnan Wosen Biotechnology Company. The antigen had a concentration of 0.236 mg/ml and purity of greater than 99.0% by h**igh-performance liquid chromatography **(HPLC) and silver staining. LPS level in the antigen was less than 10 EU/ml by Limulus assay.

### HB vaccine

The awd2 HB vaccine was made with alum adjuvant and provided by Yunnan Wosen Biocenology Company. The concentrations of HBsAg and alum were 24 μg/ml and 0.5 mg/ml respectively.

### Reagent for the detection of cytokine expression, anti-HBs and leucocyte surface molecules

The reagents for cytokine detection were provided by Panomics Company and used per protocol of each cytokine. The Anti-HBs was detected with automated chemiluminescent microparticle immunoassay, Abbott ARCHITECT^® ^anti-HBs. The reagents for the detection of leucocyte surface molecules were provided by BioLegend Company.

### Evaluation of cytokine mRNA

Splenocytes were isolated from 6- to 8-week-old female BALB/c mouse (H-2^d^, National Institute for the Control of Pharmaceutical and Biological Products, Beijing, China). Single cell suspensions (1 × 10^6^/ml) were prepared with NycoPrep ™ 1.077A (AXIS-SHIELD PoC AS, Oslo, Norway) and suspended in 1640 medium (RPMI 1640, Hyclone) with penicillin-streptomycin (final concentrations of 100 U/ml and 100 μg/ml respectively) (Sigma, Irvine, U.K.). 0.1 ml of the single cell suspension and 0.1 ml BW006 (final concentration of 5 μg/ml) were added to round-bottom 96 wells microtiter plates and cultured at 37°C with 5% CO_2_. The mRNA expression of IL-2, IL-4, IL-5, IL-10, IL-12a, IL-12b and IFN-r were analyzed at 1, 3, 6 and 12 hours respectively, each in triplicate.

### Evaluation of in vivo HBsAb production

100 6- to 8-week-old female BALB/c mice (H-2^d^, National Institute for the Control of Pharmaceutical and Biological Products, Beijing, China) were evenly divided into 10 groups and respectively received a single intramuscular i**njection **(i.m.) into left tibialis anterior muscles, of 0.1 ml solution containing 50 μg alum, 20 μg BW006, 2 μg recombinant HBsAg, 2 μg recombinant HBsAg with 1.25 μg BW006, 2 μg recombinant HBsAg with 5 μg BW006, 2 μg recombinant HBsAg with 20 μg BW006, 2 μg recombinant HB vaccine, 2 μg recombinant HB vaccine with 1.25 μg BW006, 2 μg recombinant HB vaccine with 5 μg BW006 and 2 μg recombinant HB vaccine with 20 μg BW006. The duplicate aliquots of plasma were collected weekly from 1-4 weeks, one for HBsAb testing and the other one for HBsAb titration if it was HBsAb positive. The antibodies against HBsAg were detected and quantified in triplicate for each specimen with Abbott ARCHITECT^® ^anti-HBs reagent as described above. The average value of the triplicate equal to or greater than 10 mIU/ml was considered as positive HBsAb conversion. The numbers of serum HBsAb conversion and titer of each positive serum were calculated.

### Detection of leucocyte surface molecules CD80 and CD86

Sixteen 6- to 8-week-old female BALB/c mice (H-2^d^, National Institute for the Control of Pharmaceutical and Biological Products, Beijing, China) were evenly divided into 4 groups and given a single subcutaneous injection of 0.1 ml solution containing 4 μg recombinant HBsAg, 20 μg BW006, 4 μg recombinant HBsAg with 20 μg BW006, or NS, respectively. The mice of all four groups were euthanized 12 hours after the injection and dendritic cells were separated by auto-MACs kit (Miltenyibiotec Com.) per manufacturer's instructions. Single cell suspensions (1 × 10^6^/ml) were prepared with NycoPrep ™ 1.077A (AXIS-SHIELD PoC AS, Oslo, Norway). Cell concentration of dendritic cells were adjusted to 1 × 10^5^/ml and co-culvated with rat-anti mouse APC-CD11c, CD80-FITC and CD86-PE antibodies from BioLegend company at room temperature for 20 minutes. The cells were centrifuged at 400 g for 10 minutes and re-suspended with 500 μl of 10% paraformaldehyde. The expression levels of the surface molecules CD80 and CD86 were detected and quantified by flow cytometry.

### Statistical analysis

Statistical analysis was performed by SAS version 9.2 (SAS Institute Inc., Cary, North Carolina, USA). Categorical variables were compared by Chi's square test. If 50% of cells had expected counts less than 5, Fisher's Exact test results would be used. Continuous variables were expressed as mean with standard deviation and compared by Student's t-test or Mann Whitney U test as appropriate. All statistical tests were two-sided, and p values less than 0.05 were considered statistically significant.

## Results

### Enhancement of HBsAb production

**Figure 1:**
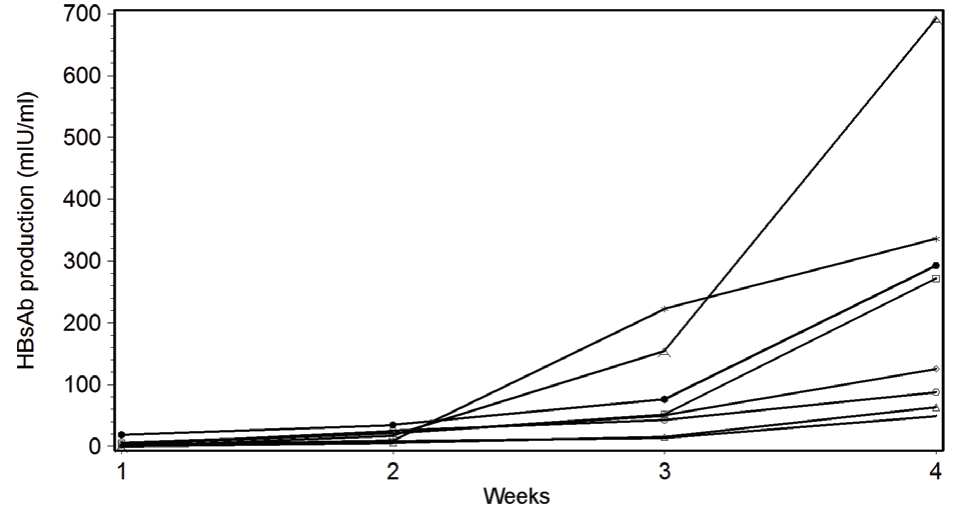
**The average production of HBsAb (mIU/ml) in mice immunized with different dosage of CpG DNA and recombinant expressed HBsAg or HBV commercial vaccine in four weeks**. A: mice immunized with HBV vaccine and 20 μg CpG DNA; star: mice immunized with HBV vaccine and 5 μg CpG DNA; dot: mice immunized with HBsAg and 5 μg CpG DNA; square: mice immunized with HBsAg and 20 μg CpG DNA; diamond: mice immunized with HBV vaccine and 1.25 μg CpG DNA; circle: mice immunized with HBsAg and 1.25 μg CpG DNA; triangle: mice only immunized with HBV vaccine; line only: mice only immunized with HBsAg; mice injected with alum and saline solution did not produce HBsAb.

HBsAb serum conversion was confirmed after 3 weeks of vaccination in all 10 mice injected with 20 μg BW006 (CpG DNA) and HBV vaccine, while 6 and 8 mice respectively had HBsAb serum conversion after 3 and 4 weeks of vaccination with regular HBV vaccine alone. The positive proportion of HBsAb serum conversion was significantly higher after two weeks of vaccination in the group vaccinated with 20 μg BW006 and HBV vaccine than that in the group with HBV vaccine alone (7 Vs. 1 out of 10, P = 0.0198). BW006 dose dependant co-stimulation effect of HBsAb serum conversion on HBV vaccine was seen between the ranges of 1.25 μg to 20 μg. The result showed that 5 μg BW006 had the best co-stimulation effect of HBsAb serum conversion for mice vaccinated with recombinant expressed HBsAg. All 10 mice vaccinated with HBsAg and 5 μg BW006 had anti-HBs serum conversion after 3 weeks. Surprisingly, 5 μg BW006 with HBsAg had almost the same co-stimulation effect on HBsAb serum conversion as 20 μg BW006 with HBV vaccine in the mouse model. However, the fourth week HBsAb concentration was at least 2 times higher in the mice vaccinated with 20 μg BW006 with vaccine than that in any other combination (Figure 1).

### Increase of cytokines mRNA production of splenocytes with stimulation of BW006

**Figure 2:**
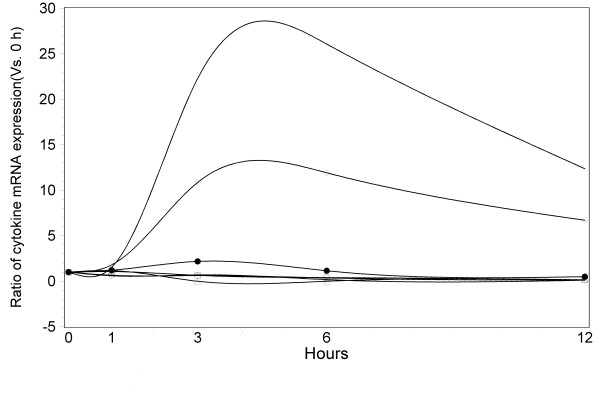
**The ratios of IL-2, IL-4, IL-5, IL-10, IL-12a, IL-12b, IFN-r mRNA expression in mouse mononuclear cells stimulated with CpG DNA (5 μg/ml) in 12 hours**. Line with squares: IL-12b; triangles: IL-10; dots: IL-12a; circles: IL-2; stars: IL-4; diamonds: IL-5; line only: IFN-r.

Splenocytes co-cultured with 5 μg/ml final concentration of BW006 had different effects on cytokines production (Figure 2). The highest increase of cytokines was IL-12b between 3 and 6 hours after the introduction of BW006 (22.29 and 26.07 times vs. 0 hour, P < 0.0001, respectively). The peak increase of IL-12b began decreasing after 6 hours of co-culture with BW006 and had almost half the peak value of mRNA at 12 hours. IL-12a increased significantly after 2 hours of stimulation. However, the mRNA production was only 2.17 times high as the value before co-culture with BW006 and decreased to the original value around 6 hours. The other significant increase of cytokine mRNA was observed in IL-10, which had a peak increase between 3 and 6 hours (10.86 and 11.93 times vs. 0 hour, P < 0.0001, respectively) and decreased to almost half at 12 hours. All the other cytokines tested, including IL-2, IL-4, IL-5 and IFN-r, had variations during the observation period. But these differences were not significant.

### Stimulation of leucocyte surface molecules CD80 and CD86

**Figure 3:**
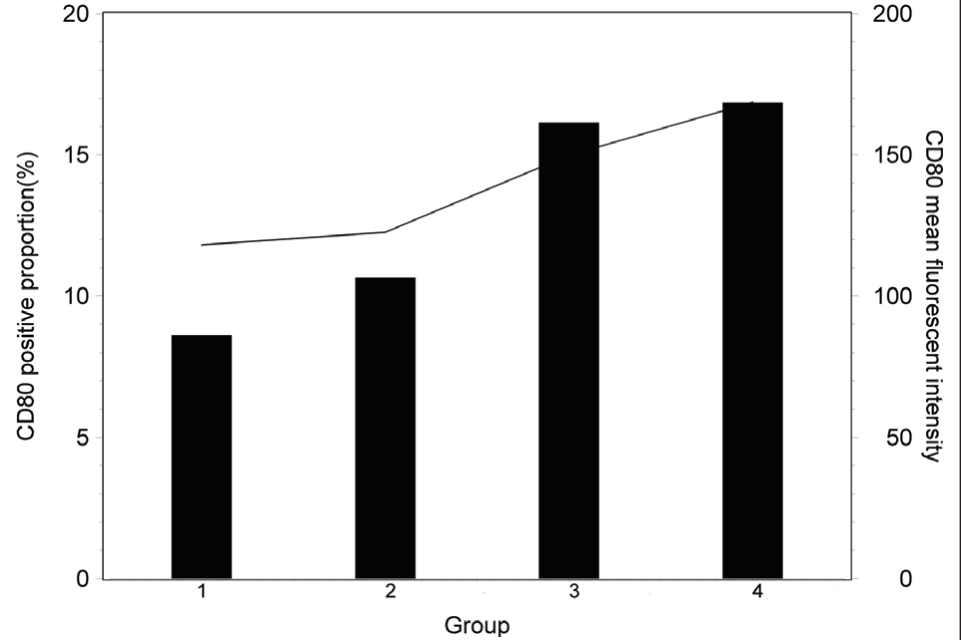
**The positive proportions (%) of CD80 (bar) and the average fluorescent intensities (line) of mouse DC cells stimulated with saline solution (group 1), 4 μg HBsAg (group 2), 20 μg CpG DNA (group 3) and 4 μg HBsAg+20 μg CpG DNA (group4), respectively**.

There was no significant difference between mice groups of negative control and 4 μg recombinant HBsAg injection in the positive proportion (8.61% Vs. 10.65%) and fluorescent intensity (118.12 Vs. 122.6) of surface molecule CD80 expression in leucocyte cells (Figure 3). 20 μg BW006 increased the positive proportion (15.14% for BW006 alone and 15.84% for BW006 and 4 μg recombinant HBsAg) and fluorescent intensity (139.86 for BW006 alone and 158.67 for BW006 and 4 μg recombinant HBsAg) of surface molecule CD80 expression in leucocyte cells.

**Figure 4:**
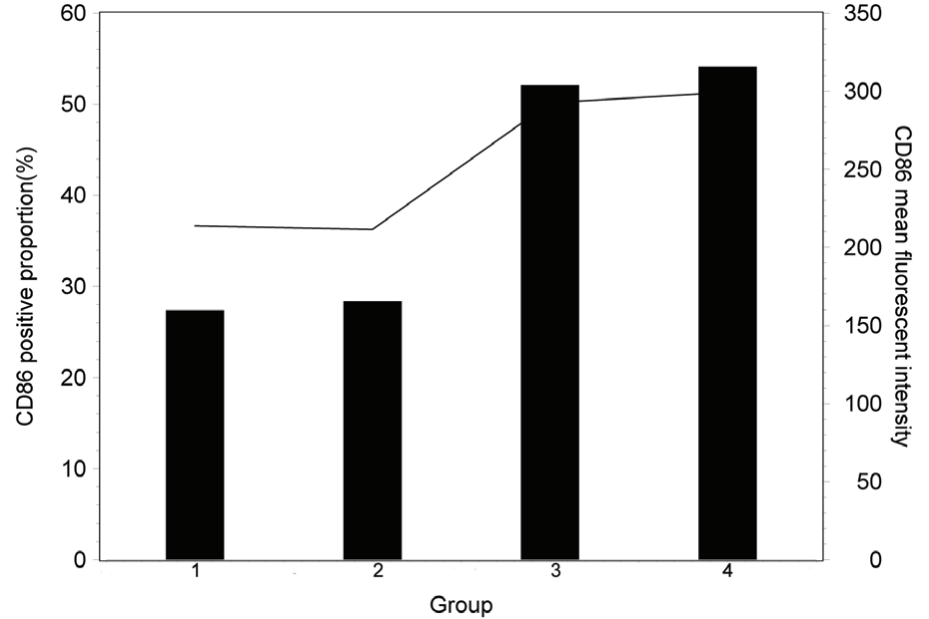
**The positive proportions (%) of CD86 (bar) and the average fluorescent intensities (line) of mouse DC cells stimulated with saline solution (group 1), 4 μg HBsAg (group 2), 20 μg CpG DNA (group 3) and 4 μg HBsAg+20 μg CpG DNA (group 4), respectively**.

The same trend was seen in CD86 (Figure 4), in which positive expression proportion was almost doubled in leucocyte cells compared with that in the control group (52.12% vs. 27.37%) or in 4 μg recombinant HBsAg injection alone group (54.09% vs. 28.36%). The fluorescent intensity of surface molecule CD86 expression also increased with BW006 compared with that of the control group (292.68 Vs. 213.78) or of HBsAg alone group (299.35 Vs. 211.78) in leucocyte cells.

## Discussion

Alum adjuvant HBV vaccine has been used for hepatitis B prevention for decades. However, there have been vaccine unresponsive cases reported in the immunodeficient patients of uremia and other conditions [11-13]. Scientists have been working for applicable new adjuvant for HBV and other diseases [14-17]. CpG ODN can enhance the immune response of live-attenuated or multivalent vaccines that cannot mix with alum. There have been studies that confirmed the potentiality of CpG ODN as HBV adjuvant [10,18]. We designed the new sequence of CpG ODN with two motifs that could improve the immune response of HBV recombinant expressed HBsAg and also vaccine (containing alum adjuvant). The co-stimulatory effect was observed dynamically with anti-HBs production in mice. The production of cytokines in vitro and the expression of leucocyte surface molecules CD80 and CD86 were evaluated in vitro for the possible mechanism of the enhanced immunization of HBV.

Our new CpG ODN alone can induce HBsAg producing anti-HBs response or together with alum in the mouse model (CpG ODN and regular vaccine groups). However, a stronger specific antibody production was observed in the mice immunized with CpG ODN and HBV regular vaccine after 3 weeks of vaccination, owing to the synergistic action of CpG and alum. Alum adjuvant has an important disadvantage of induction of a Th2 rather than a Th1-type immune response. The use of alum as an adjuvant was reported to interfere with cell-mediated immunity and blocks activation of CD8+ CTL [19]. The Th1 immune response induced by CpG ODN was able to overcome the Th2 bias of alum for both antibody isotype and CTL response when both the agents were used together [20]. Our results showed even higher specific antibody production.

Interleukin-12, a cytokine with an important role against intracellular pathogens, promotes Th1 cell development, cell mediated cytotoxicity, and interferon-gamma production. The production of IL12, especially the increase of IL-12b played an important role in HBV clearance [21]. Almost a 30-fold increase of IL12b was observed in splenocytes after 3-6 hours co-culture with 5 μg/ml final concentration of CpG ODN. The significant increase of IL10 seems to play a role in Th1/Th2 cells balance.

The T cell immune response requires co-stimulatory signals delivered through one or more receptors on the surface of T cells. The proteins, CD80 and CD86 (also known as the B7-1 and B7-2 ligands), are molecules found on activated B cells and monocytes, which provide a costimulatory signal necessary for T cell activation and survival [22,23]. The expression of CD80 and CD86 has been used for evaluation of T cell response for vaccine development [24-26]. The increased production of HBsAb as well as IL12 and IL10 might be associated with the activation of CD80 and CD86 expression in the experiment groups with HBsAg and CpG-ODN. Decreased function of peripheral blood dendritic cells has been reported in patients with hepatocellular carcinoma with hepatitis B and C virus infection [27]. Myeloid dendritic cells (mDC) of patients with chronic HBV are impaired in their maturation and function, resulting in more tolerogenic rather than immunogenic responses, which may contribute to viral persistence [28]. It is interesting that there was no significant difference in positive proportion or intensity of CD80 and CD86 expression between the mouse group vaccinated with CpG-ODN alone and the group with CpG-ODN together with recombinant HBsAg in our experiment.

## Competing interests

The authors declare that they have no competing interests.

## Authors' contributions

XZ and PH contributed equally to the manuscript. XZ and PH carried out the mouse vaccination, the detection of HBsAb, cytokines and the surface molecules CD80 and CD86 production. ZH assisted the immunoassays. XW performed the statistical analysis and drafted the manuscript. ZL conceived of the study, and participated in its design and coordination. All authors read and approved the final manuscript.
